# Molecular pathways leading to loss of skeletal muscle mass in cancer cachexia – can findings from animal models be translated to humans?

**DOI:** 10.1186/s12885-016-2121-8

**Published:** 2016-02-08

**Authors:** Tara C. Mueller, Jeannine Bachmann, Olga Prokopchuk, Helmut Friess, Marc E. Martignoni

**Affiliations:** Department of Surgery, Klinikum rechts der Isar, Technische Universität München, Ismaninger Strasse 22, D-81675 Munich, Germany

**Keywords:** Cancer cachexia, Skeletal muscle wasting, Signaling pathways, Targeted therapies, Animal models

## Abstract

**Background:**

Cachexia is a multi-factorial, systemic syndrome that especially affects patients with cancer of the gastrointestinal tract, and leads to reduced treatment response, survival and quality of life. The most important clinical feature of cachexia is the excessive wasting of skeletal muscle mass. Currently, an effective treatment is still lacking and the search for therapeutic targets continues. Even though a substantial number of animal studies have contributed to a better understanding of the underlying mechanisms of the loss of skeletal muscle mass, subsequent clinical trials of potential new drugs have not yet yielded any effective treatment for cancer cachexia. Therefore, we questioned to which degree findings from animal studies can be translated to humans in clinical practice and research.

**Discussion:**

A substantial amount of animal studies on the molecular mechanisms of muscle wasting in cancer cachexia has been conducted in recent years. This extensive review of the literature showed that most of their observations could not be consistently reproduced in studies on human skeletal muscle samples. However, studies on human material are scarce and limited in patient numbers and homogeneity. Therefore, their results have to be interpreted critically.

**Summary:**

More research is needed on human tissue samples to clarify the signaling pathways that lead to skeletal muscle loss, and to confirm pre-selected drug targets from animal models in clinical trials. In addition, improved diagnostic tools and standardized clinical criteria for cancer cachexia are needed to conduct standardized, randomized controlled trials of potential drug candidates in the future.

## Background

Cancer cachexia is a multi-factorial, systemic syndrome that occurs in the course of malignant diseases, especially in cancer of the gastrointestinal tract (GIT) [1, 2]. When all types of cancer are considered, cachexia affects around 60 % of patients in the course of their disease [3]. In gastric or pancreatic cancer even 80 % of patients are affected [1, 2, 4, 5]. In addition, cachexia is also observed in the course of benign diseases like chronic heart failure, renal failure and chronic obstructive pulmonary disease (COPD). The cachexia syndrome is characterized by weight loss due to excessive wasting of skeletal muscle and adipose tissue mass, which usually cannot be reversed by conventional nutritional support and is frequently accompanied by anorexia, fatigue, anemia and abnormal metabolism [2, 6, 7]. In cancer patients, cachexia can occur in every stage of disease and is associated with a poor prognosis, reduced treatment tolerance and a marked reduction in quality of life (QoL). The diagnostic criteria for cancer cachexia are weight loss >5 % or weight loss >2 % in individuals with a body mass index (BMI) <20 kg/m^2^ over 6 months in absence of simple starvation, or the presence of sarcopenia (skeletal muscle index <7.26 kg/m^2^ for males and <5.45 kg/m^2^ for females) with any degree of weight loss >2 % [8]. Furthermore, reduced food intake, anorexia, markers of systemic inflammation like C-reactive protein (CRP), responsiveness to chemotherapy and the rate of cancer progression should be assessed for the diagnosis of cancer cachexia [8]. The presence of cachexia in cancer patients is associated with reduced treatment response and tolerance and accounts for at least 20 % of cancer-specific mortality [2, 4, 9]. Furthermore, cachectic patients have an elevated surgical risk. Therefore, preservation of lean body mass can be critical for the survival of cancer patients, but an effective treatment for cachexia is still lacking.

Skeletal muscle wasting is the most important phenotypic feature of cancer cachexia and is among the principle causes of functional impairment, respiratory complications and fatigue [10, 11]. A recent study on cachectic patients with cancer of the GIT showed that besides the loss of muscle mass there is a substantial loss of muscle strength and mechanical quality [11]. In contrast to starvation, the non-muscle protein compartment of the body remains relatively unaffected and the liver mass is even increased, implying a tumor-associated metabolic condition that specifically targets skeletal muscle and subcutaneous adipose tissue [7]. Even though pathophysiological mechanisms of the depletion of skeletal muscle tissue during cancer cachexia have been intensely studied in recent years, identification of the key processes and therapeutic targets has been impeded by the large number of mediators and signaling pathways involved [12]. Furthermore, there is evidence of complex tissue interactions in this systemic syndrome, mediated through cytokines, tumor-derived factors, hormones and neuropeptides [1, 6, 12, 13]. However, so far the majority of studies have been conducted in animal models and studies of human muscle biopsies remain scarce and inconsistent [14]. The aim of this review is to outline these discrepancies and investigate to which degree findings from animal studies are translatable into clinical practice and research.

## Mediators and signaling pathways in cancer cachexia – findings from animal models vs. human samples

Cancer cachexia is a complex systemic syndrome that involves a large number of systemic pro- and anti-inflammatory mediators as well as hormones, neuropeptides and tumor derived factors. However, in the following only those systemic mediators and pathways, on which sufficient comparable studies in animals as well as humans were available, will be first reviewed and then discussed. Table 1 gives an overview of these mediators and mechanisms and the findings regarding their role in animal *vs.* human studies.

**Table 1:** Mediators and mechanisms of skeletal muscle loss in cancer cachexia: correlation of findings in animal models and humans

	Animal models	Humans
TNF-α	++	+/−
	Yoshida hepatoma/sarcoma, LLC, Leydig cell tumor, Morris hepatoma	Various types of solid tumors
TRAF-6	+	+
	LLC	Gastric cancer
IL-6	++	+/−
	C26, Morris hepatoma, ApcMin/+	Various types of solid tumors
IL-1	+	+/−
	Methylcholanthrene-induced Sarcoma, Prostate ADK	Various types of solid tumors
INF-γ	+	+/−
	MAC16	Various types of solid tumors
Myostatin/ TGF-ß	++	+
	C26, MAC16	Gastric cancer
PIF	+	+/−
	MAC16	GIT cancers
Angiotensin II	+	+
	C26	NSCLC, congestive heart failure
Ubiquitin-Proteasome system	++	+
	C26, Yoshida hepatoma, LLC	GIT cancers
Autophagy-lysosomal system	+	+
	C26, Yoshida hepatoma, LLC	Lung cancer
IGF-1/Pi3K/Akt/mTOR	+/−	+/−
	C26, ApcMin/+	Various types of solid tumors
MRFs (Myo D, Pax7)	+	+
	C26	Pancreatic cancer

+ role confirmed in few studys

++ role confirmed in many studys

+/− role not confirmed/inconsistent results

Abbreviations: *TNF* tumor necrosis factor, *LLC* Lewis lung cell carcinoma, *TRAF* TNF-receptor adaptor protein, *IL* interleukin, *INF* interferon, *TGF* transforming growth factor, *PIF* proteolysis inducing factor, *IGF* insulin like growth factor, *MRF* muscle growth and regeneration factor

### Cytokines

#### TNF-α, TRAF6

Tumor necrosis factor (TNF)-α and TNFR-1 mRNA were shown to be elevated in several animal models of cancer cachexia and pharmacological inhibition of TNF-α showed a reduction in weight loss due to cancer in rodents [15–21]. As was recently reviewed by Baracos et al., TNF-α especially seems to play a role in the Yoshida hepatoma and sarcoma rat model as well as the Lewis lung carcinoma (LLC) model, but not in the C26- or MAC16- adenocarcinoma mouse models [22]. Recently, TNF-α receptor adaptor protein 6 (TRAF6) [23–26] which functions as a E3 ubiquitin ligase, has also been shown to be involved in catabolic signaling of cachexia in LLC mice [25].

In humans, several studies also found correlations of TNF-α serum levels with cachexia. A study on patients with pancreatic cancer showed that serum TNF-α levels were inversely correlated with BMI, hematocrit, hemoglobin, serum protein and albumin levels [27] and similar observations were made in patients with prostate cancer [28, 29] and hepatocellular carcinoma [30]. In addition, it was shown that expression of the TNF-α gene was upregulated in patients with pancreatic cancer and normalized after the tumor was surgically resected [31]. Others observed significant differences in serum TNF-α of patients and controls, but no correlation with weight loss [32, 33]. Interestingly, a recent study on 102 gastric cancer patients showed that TRAF6 mRNA and protein, as well as ubiquitin mRNA and protein, were all upregulated in skeletal muscle tissue and correlated with disease stage and the degree of weight loss. The positive correlation between TRAF6 and ubiquitin expression suggests that TRAF6 may regulate ubiquitin activity in human cancer cachexia [34].

#### Interleukin-6

Another pro-inflammatory mediator with a critical role in muscle wasting during cancer cachexia is interleukin (IL)-6 [35]. Elevated serum IL-6 levels have been observed in C26 and Apc^Min/+^ mouse models of cancer cachexia, and systematic administration of IL-6 to these mice resulted in depletion of skeletal muscle and adipose tissue and ultimately led to death. Furthermore, pharmaceutical inhibition of IL-6 signaling was shown to decrease the rate of cachexia in tumor-bearing rodents [35–39]. In skeletal muscle, the three most important intracellular signaling pathways induced by the ligand-receptor binding of IL-6 are the activation of JAK/STAT3, ERK and PI3K/Akt pathways [40–42]. In vitro tests have shown that the activation of STAT3 is both necessary and sufficient to induce muscle wasting. Apc^Min/+^ mice also showed increased activation of STAT3 in skeletal muscle [37]. Pharmacological inhibition of STAT3 was able to reduce muscle atrophy in mice with colon carcinoma; however, it was not sufficient to completely attenuate cachexia [40].

In human studies, elevated serum IL-6 levels were quite consistently associated with weight loss and a reduced rate of survival in cancer patients [1, 35, 40, 43–51]. Moreover, IL-6 was shown to be significantly over-expressed in pancreatic cancer tissue, and serum levels were significantly elevated in cachectic compared to non-cachectic patients with pancreatic cancer [48, 52, 53] and prostate cancer [49].

#### IL-1β and INF-γ

In some animal models, IL-1 and interferon (INF)-γ were shown to induce weight loss and anorexia, and neutralizing IFN-γ antibodies successfully attenuated cachexia [16]. In particular IL-1ß appears to be essentially involved in the central regulation of food intake and feeding behavior [54]. In a study of patients with advanced upper GIT cancer or NSCLC, IL-1ß was shown to be a better predictor of cachexia than IL-6, which did not correlate with weight loss in this study population [44]. In another study on GIT cancer patients, a correlation between weight loss and serum vascular endothelial growth factor (VEGF)-A, as well as between VEGF-A, IL-6 and IL-1 serum levels were observed [32, 33]. However, there are also several studies that did not find any correlation of serum cytokine levels with weight loss or cachexia in cancer patients [55–57].

### Increased proteolysis

#### Myostatin

Mediators of increased proteolysis in cancer cachexia include members of the transforming growth factor (TGF)-β family. Myostatin is a secreted protein expressed predominantly in skeletal muscle and to a lesser extent in cardiac muscle and adipose tissue [58–60]. It has recently been shown that myostatin is also secreted by C26 carcinoma cells and other murine and human neoplasms [61]. Free myostatin binds to a high-affinity activin type-2 (ActRIIB) receptor in skeletal muscle, which induces a range of intracellular signaling cascades leading to increased proteolysis [4, 58, 59, 61, 62], as well as the inhibition of anabolic pathways (IGF-1/Akt) [63–65] and muscle regeneration [59, 61, 62, 66]. In a murine model of cancer cachexia, inhibition of myostatin by specific antibodies was able to attenuate the atrophy of skeletal muscle and improved muscle mass and function [67, 68]. In addition, blocking of the ActRIIB receptor attenuated the wasting process in skeletal muscle and heart and was associated with increased survival of tumor-bearing mice in several models of cancer cachexia [13, 58, 69]. Furthermore, this treatment brought elevated serum levels of myostatin back to normal and attenuated cachexia, independently of pro-inflammatory cytokine levels [70]. Other TGF-ß family members that act through the ActRIIB receptor are activin A, inhibin and macrophage inhibitory cytokine-1 (MIC-1/GDF-15) [58]. In mouse models, animals given either myostatin or activin A showed up to a 30 % decrease in muscle mass [13, 58, 71]. Furthermore, the inhibition of activin A was able to rescue myoblasts treated with TNF-α or IL-1 and allowed normal differentiation into myotubes [72]. In addition, activin A signaling has been linked to increased mitochondrial energy metabolism and oxygen consumption, supporting its role in maintaining body weight and resting energy expenditure (REE) [73]. Treatment with a specific anti-activin A antibody was able to prevent cachexia and death in a mouse model [13, 70]. Another member of the TGF-ß family, Inhibin is a secreted tumor suppressor and is a competitive antagonist of activin for the ActRIIB receptor. Inhibin deficiency causes gonadal tumor growth and severe cachexia in animals. Treatment of inhibin-deficient mice with an ActRIIB antibody prevented cachexia, reduced tumor growth, and prolonged survival [74, 75]. Moreover, it has recently been shown that MIC-1 over-expressing tumor-bearing mice showed decreased food intake and increased loss of muscle and fat mass. The degree of serum MIC-1 elevation directly correlated with the amount of cancer-related weight loss, which was reversed by neutralization of MIC-1 with a specific monoclonal antibody [76].

Studies investigating the relevance of myostatin or ActRIIB signaling in human cancer patients are scarce. Aversa et al. observed that myostatin was significantly increased in patients with gastric cancer who did not lose weight, whereas Smad2 expression was unchanged. Interestingly, in the same study, lung cancer patients had no increase in myostatin serum levels but did have increases in Smad2 expression, raising the question whether different tumors might induce different patterns of molecular changes within skeletal muscle tissue [77].

#### Proteolysis-inducing factor

A much-debated mediator of increased proteolysis and muscle loss in cancer cachexia is the tumor derived proteolysis-inducing factor (PIF). In experimental cancer cachexia, PIF has been shown to induce degradation of skeletal muscle protein via the ubiquitin proteasome system (UPS) [78–84]. Proteolysis-inducing factor was first isolated from the urine of tumor-bearing mice, and was shown to induce the increased expression of proteasome subunits and increased proteasome activity via NF-κB [82, 85–87]. The activation of NF-κB includes the phosphorylation of RNA-dependent protein kinase (PKR), which inhibits protein synthesis [78]. The relevance of this process to cancer cachexia is demonstrated by the ability of a PKR-inhibitor to abate skeletal muscle atrophy in a mouse model of cachexia by attenuating UPS-dependent proteolysis and increasing protein synthesis [88].

However, in human cancer cachexia, the existence of a homologue to PIF is controversial [86, 89]. First, a homologue glycoprotein was isolated from urine of weight-losing cancer patients, and when purified and injected into mice, it induced a 10 % loss of body weight within 24 h [54]. Subsequently, several studies showed an association between PIF and weight loss in patients with cancer cachexia [90–93]. Immunohistochemical staining of tumor samples from patients with GIT cancers showed that PIF was expressed by the tumor, which was strongly associated with weight loss and the presence of PIF in urine samples [91]. However, the only longitudinal study on the presence of PIF in urine of 36 GIT cancer patients showed that over time, cancer patients positive for the PIF pattern experienced weight loss, whereas those with a negative test gained weight [92]. Based on the available sequence of PIF, the factor HCAP (Human cachexia-associated protein) was identified in cell lines, in metastatic tumors and in the urine of cancer patients with cachexia [93]. In addition, the same research group showed that the expression of HCAP in prostate cancer cells was associated with disease progression and the development of cachexia [93]. In contrast, other groups found that the presence of PIF in urine did not correlate with weight loss, anorexia, tumor response, or survival in cancer patients [89, 94].

#### Angiotensin II

Similar to the action of PIF, angiotensin-II (AngII) has been shown to directly accelerate protein breakdown by the UPS in vitro [95]. In animals, it was shown that proteolytic system compounds were upregulated in skeletal muscle by AngII, and the subsequent increase in protein degradation was blocked by muscle-specific expression of IGF-1 [95–97]. The action of AngII involves activation of NF-κB-dependent signaling and the formation of reactive oxygen species (ROS) [98]. In mice with cancer cachexia, inhibition of ROS formation by the antioxidant α-tocopherol was able to rescue skeletal muscle mass [80, 99]. Furthermore, in a recent study on the effect of treatment of C26-mice with angiotensin-converting enzyme (ACE) inhibitors, it was shown that muscle mobility and strength, as well as respiratory function were improved although body and muscle mass were not increased [100].

However, the role of AngII in human cancer cachexia remains to be determined. In patients with cachexia related to congestive heart failure, treatment with ACE inhibitors caused an increase in both subcutaneous fat and muscle mass [101]. There is also some preliminary evidence that ACE inhibitors have the potential to ameliorate cancer cachexia, at least in NSCLC patients [102]. In addition, treatment with antioxidants has been shown to be effective in increasing lean body mass, decreasing ROS and pro-inflammatory cytokines, and improving QoL [103].

#### Activation of proteolytic systems

In experimental cancer cachexia the ubitquitin-proteasome-system (UPS) has been shown to play the major role in the degradation of muscular proteins [1, 15, 104, 105]. Increased mRNA levels of ubiquitin and proteasome subunits, as well as increased proteasome activity, have been observed in numerous animal models of cancer cachexia [18, 38, 39, 105–110]. Treatment with proteasome inhibitors was successful in ameliorating cachexia in C26-mice [111]. In animal models the regulation of expression of genes for the E3-ubiquitin ligases, muscle ring finger-1 (*MuRF-1*) and muscle atrophy F box (*MAfxb*) has been shown to be increased in different types of muscle atrophy, including cancer cachexia [112–120]. *MuRF-1* and *MAfxb* expression in skeletal muscle are regulated by transcription factors of the FoxO family [38, 62, 112, 113, 121–124] and NF-κB [87, 125]. Furthermore, the activation of the transcription factors Smad2/3 (myostatin pathway) also increases the expression of *MuRF-1* and *MAfxb* via FoxO [4, 58, 59, 61, 62, 104, 113, 121]. Inhibition of *MAfxb* in fasting mice with muscular atrophy led to decreased expression of myostatin and increased expression of the transcription factor MyoD which is implicated in muscle regeneration [126]. Administration of IL-6 to *Apc*^*Min/+*^ mice induced *MAfxb* expression via STAT3, whereas *MuRF-1* was unaltered at gene and protein levels [35]. These findings suggest that *MuRF-1* and *MAfxb* are involved in different catabolic signaling pathways and represent a common effector.

Compared to these results from animal models, studies of human skeletal muscle have so far produced rather ambivalent results regarding the activation of the UPS in cancer cachexia. Increased activity of the UPS in correlation with disease severity has been demonstrated in skeletal muscle of patients with gastric cancer [34, 127–129], even before the clinical onset of cachexia [128, 129]. Accordingly, it was observed that markers of systemic inflammation (IL-6, CRP) correlated with the increased expression of ubiquitin in skeletal muscle biopsies from cachetic patients with pancreatic cancer, and this increase correlated with the degree of weight loss [130]. Muscle proteolysis and proteasome subunit mRNA were also elevated in colorectal cancer patients compared to controls, whereas after surgical resection of the tumor, the proteolysis rate was reduced to the level of healthy controls [131]. However, a study on patients with pancreatic cancer showed that components of the UPS were only upregulated in subjects with weight loss >10 % [132]. In contrast, studies on lung cancer patients did not find any increased expression of UPS components in skeletal muscle biopsies [133, 134]. Other studies observed that *MAfxb* expression and *MuRF-1* expression were unaltered in muscle biopsies of patients with gastric [135] and colorectal cancer [131]. Interestingly, a study on pancreatic cancer patients with weight loss found that the expression of *FoxO1* and −*3* was even decreased in skeletal muscle biopsies compared to controls [136].

Similarly, studies investigating the activation of NF-κB in human cancer cachexia have also produced inconsistent results. Whereas the activation of NF-κB has been shown to be an early and sustained event in patients with gastric cancer [137], pre-cachetic lung cancer patients did not show any activation of muscular NF-κB-dependent inflammatory signaling [134]. Furthermore, two recent studies on gene expression profiles in human cancer cachexia showed that none of the previously described genes, including *MuRF-1*, *MAfxb* and autophagy related genes (Atgs), were upregulated in skeletal muscle biopsies [138, 139].

Protein-degradation via the authophagy-lysosomal system (ALS) is getting more and more attention in the context of cancer cachexia recently, and its regulation has been shown to overlap with the UPS. Under physiological conditions, the ALS contributes to cell survival and adaption to stress through the controlled degradation of dysfunctional intracellular organelles and proteins by lysosomal proteases (cathepsins) [140]. The regulation of autophagy induction and autophagosome formation is dependent on gene expression of *Atgs*. In animal models, *Atgs* (e.g. *Atg7*, *LC3B*, *Bnip3*, *Beclin-1*) have been shown to be over-expressed during muscle atrophy [140–143] and are partly regulated by FoxO3 [121, 143, 144] and p38-MAPK [145, 146]. Furthermore, elevated levels of total cathepsin activity have been observed in hepatoma-bearing rats and treatment with a cathepsin-inhibitor was able to attenuate muscle depletion [147, 148]. A recent study by Penna et al. demonstrated that autophagy is increased in C26-bearing mice as well as in Yoshida-hepatoma rats and LLC mice [149].

However, in skeletal muscle of patients with early stages of lung cancer, mRNA levels of the lysosomal proteases cathepsin B and D were elevated, whereas components of the UPS were not increased [133]. Furthermore, cathepsin B mRNA levels correlated with fat-free mass index and tumor stage and were higher in cancer patients who were smokers. Since these observations were made before the clinical onset of cachexia, it is suggested that similar to findings from animal models, cathepsin B expression is involved in the induction of cachexia in lung cancer patients [133]. Increased levels of cathepsin D have been observed in cancer patients with other tumor entities as well [150].

### Decreased protein synthesis

In addition to the increased protein degradation, it has been postulated that muscle atrophy in cancer cachexia is also due to decreased protein synthesis [78, 141]. Under physiological conditions, activation of the anabolic PI3K/Akt/mTOR pathway results in down-regulation of *MuRF-1* and *MAfxb* through inhibition of FoxO [12, 119, 136] and simultaneous stimulation of protein synthesis by activation of mammalian target of rapamycin (mTOR) and glycogen synthase kinase 3β (GSK3β) [12, 119, 136, 151]. The PI3K/Akt/mTOR signaling cascade is activated by insulin or IGF-1. Low levels of insulin or IGF-1 and elevated levels of glucocorticoids induce the loss of muscle protein in diabetes, and insulin resistance is a characteristic feature of many systemic diseases with muscle wasting [152].

Activation of the protein kinase Akt was shown to be decreased in muscle and adipose tissue of tumor-bearing, cachectic mice [124]. In contrast, a recent study on cancer cachexia in mice observed an increase in Akt activation, while mTOR signaling and FoxO activity were suppressed [38]. However, Penna et al. observed no decreased activity of Akt in two distinct animal models of cancer cachexia [42]. Downstream of Akt, no suppression of protein synthesis was observed, with levels of activated p70S6K and GSK3-β being normal or increased, and levels of eIF2α being decreased [42]. Other studies even found that mTOR signaling was activated rather than suppressed during cancer cachexia. One hypothesis is that mTOR may be activated through the intracellular concentration of free amino acids, which rises when protein degradation is increased [153]. According to another hypothesis a certain increase in protein synthesis is required for protein degradation, and the PI3K/Akt/mTOR pathway has a dual role. When mTOR was inhibited 30 min before the application of PIF, protein degradation was not increased, suggesting that increased proteolysis requires the activation of mTOR [85]. In addition, Robert et al. showed that beyond stimulating the synthesis of muscle protein, mTOR also regulated the production of pro-cachetic factors such as IL-6 and IL-10, and inhibition of mTOR with rapamycin resulted in reduced IL-10 mRNA translation and ameliorated the cachectic phenotype [154].

Available data on the role and activation status of the PI3K/Akt/mTOR pathway in cancer patients is very limited. In one study on patients with pancreatic cancer and weight loss, decreased protein levels and activation of Akt, mTOR, p70S6K, GSK3-ß and FoxO1 were observed in skeletal muscle tissue compared to samples from patients with pancreatic cancer without weight loss [136]. Another study on patients with GIT-cancer and weight loss found increased protein levels and phosphorylation of PKR and eIF2α. Myosin levels decreased as the weight loss increased. The linear relationship between myosin expression and the extent of phosphorylation of eIF2α and PKR suggests that the phosphorylation of PKR may be an important initiator of muscle wasting in cancer patients [155]. In addition, a study on patients with colorectal cancer investigated the pattern of muscle protein turnover before and after surgical tumor resection compared to healthy controls and evaluated the anabolic response of skeletal muscle tissue to nutrition in three groups (control, pre-operative, post-operative). The authors observed that myofibrillar protein synthesis increased after feeding of healthy controls, whereas there was no response in the preoperative cancer patient group. After surgery the anabolic response to feeding was recovered. However, in the healthy control group and in the preoperative patient group, nutrition led to a significant increase in p70S6K and 4E-BP1 phosphorylation, whereas in the postoperative patient group nutrition did not lead to this effect. The phosphorylation of Akt was unchanged in all groups. Furthermore, increased expression of proteasome subunit mRNA was observed in the preoperative group compared to controls, but interestingly *MuRF-1* and *MAfxb* expression were unchanged in all groups [131].

### Inhibition of muscle regeneration

Finally, the inhibition of positive regulators of muscle growth and regeneration factors (MRFs) also plays an important role in cancer cachexia. The transcription factor MyoD is an essential regulator of myogenesis and myoblast differentiation, and is crucial for regeneration of muscle tissue from satellite cells [14, 156, 157]. MyoD was shown to be inhibited by pro-inflammatory cytokines, myostatin and PIF. The proteolysis of MyoD during muscle atrophy was shown to be effectuated through ubiquitination by MAfxb in vitro and in vivo, and atrophy was attenuated by inhibition of this process [156].

Data on the impairment of MRFs in human cachexia patients is very limited. A study on the expression of genes involved in muscle regeneration (*Pax7*, *MyoD*, *Myf5*, *nMyHC, necdin*) in gastric cancer patients found that *Pax7* was significantly increased in all disease stages compared to controls, whereas *MyoD* and *necdin* were only upregulated in early disease stages [10]. *Pax7* was also shown to be dysregulated in muscle biopsies of patients with pancreatic cancer [158]. These results suggest that catabolic signals possibly stimulate a counteractive regenerative response in satellite cells. However, it is suggested that the regenerative response is dysfunctional during cancer-induced muscle wasting. Furthermore, these results show a possible protective role of the protein necdin, which has previously been shown to prevent cachexia in a mouse model [10].

## Discussion: signaling pathways leading to skeletal muscle mass in cancer cachexia – can findings from animal models be translated to humans?

### General issues in cancer cachexia research and clinical trials

The results presented in this review underline the fact that human cancer cachexia is a heterogeneous clinical syndrome with many variable contributing factors, all of which cannot be reflected by an animal model. Advantages of animal models clearly are the homogeneity of study subjects and the possibility to effectively control influencing confounders, e.g. by accurate control of diet or exercise and the use of pair fed controls [159]. However, none of the numerous animal models is ideal to simulate the complex biology behind human cancer cachexia. Too many variables are playing a role, including tumor biology and location, host-tumor interactions, co-morbidities, prior anti-cancer therapies and psychosocial issues. For example, implanted tumors in syngeneic animal models are well defined and do not metastasize, which does not accurately reflect the growth of malignant human tumors. Furthermore, the tumors are usually implanted in very young animals, which undergo rapid tumor growth up to a size of more than 10 % of the body mass and severe wasting within days to weeks. In contrast, growth and wasting are less aggressive in humans, where cachexia usually occurs within months to years and tumors usually don’t grow to more than 1 % of body mass [35, 160]. Xenograft models resemble more to human tumors, but lack of the interaction between tumor and immune system due to the needed immunosuppression of the animals. Genetically engineered animals develop tumors spontaneously and reproduce naïve tumor-host interactions, but the genetic alterations are expressed in all tissue, which is also not the case in human tumorigenesis. Carcinogen-induced tumors in animals probably reflect normal tumor growth and tumor-host interactions most closely, but are tedious and costly [54]. However, since none of the models addresses all aspects of human cancer cachexia, use of a combination is recommended, and careful consideration of these issues is needed before translation to clinical research can be made [54].

Compared to the paucity of literature on experimental cancer cachexia, studies on human samples are relatively scarce. This might be an accessibility issue, which is why the cooperation of researchers with surgical departments who can easily provide intraoperative muscle biopsies from cancer patients, should be encouraged.

Another general issue in cachexia research is the difficulty of recruiting a homogeneous study cohort of cancer patients. The clinical presentation of cachexia is becoming more and more variable in the context of the growing proportion of obese patients [12, 161]. Furthermore, skeletal muscle mass is generally greater in men than in women, but paradoxically loss of muscle and lean body mass is also greater in men than in women, which is possibly due to hormonal differences [12, 162]. There is also a great heterogeneity in energy expenditure between patients. As observed by Knox et al., cancer patients can be hypermetabolic, normal or hypometabolic [12, 160, 163]. Decreased physical activity combined with increased REE is another very individual and confounding element. Cancer patients are often prone to physical inactivity due to their age and co-morbidities. It is known that bed rest by itself causes muscle wasting by amplifying catabolism and desensitizing muscle cells for anabolic signals [161]. In addition, anorexia, reduced food intake, psychosocial issues and aggressive anti-cancer therapies contribute to weight loss and cachexia in human cancer patients [164]. Several modern anti-cancer therapies target signaling pathways which are important for tumorigenesis, such as Akt/mTOR, but also regulate protein anabolism in skeletal muscle and other tissues, so muscle wasting can probably be partially attributed to chemotherapy [161]. Finally, it has to be assumed that there is a genetic contribution to cachexia as well, since even with the same tumor type and stage, some individuals develop cachexia whereas other do not [12]. Studies trying to identify genes for a predisposition to cancer cachexia have shown that single nucleotide polymorphisms in cytokine genes have been associated with the prevalence of cachexia [12]. In addition, polymorphisms in the vitamin D receptor have been proposed as early clinical predictors of the development of a more aggressive form of cachexia in cancer patients [165]. Furthermore, the 1082G allele in the IL-10 promoter [166] and the C allele of the rs6136 polymorphism in the P-selectin gene have been validated as pro-cachectic genotypes [167]. These findings point towards the close relationship between the innate immune system and cancer cachexia. However, genome-wide studies are currently lacking, and the role of genetic predisposition has not yet been fully clarified [168].

Further problems are encountered in the design of clinical trials for cancer cachexia therapies. How to approach such studies has been extensively discussed as a result of the fact that many randomized controlled trials were conducted without resulting in any approved therapies [164]. A general consensus over the definition and use of diagnostic criteria of cancer cachexia and its different stages has still not been reached. The use of new, much more specific and sensitive measuring techniques of body composition, using computed tomography (CT) imaging and novel tracer techniques with labeled amino acids, will allow precise quantification of muscle protein kinetics and will facilitate the definition of more specific endpoints and guide the evaluation of pharmaceutical interventions [169]. A recent study comparing different diagnostic and assessment criteria for cachexia in a cohort of patients with advanced colorectal cancer found that the Cancer Cachexia Study Group’s cachexia score was the best prognostic factor for overall survival [170]. This score includes three diagnostic criteria: (1) weight loss >10 %, (2) intake <1500 kcal/d, and (3) CRP >10 mg/L. However, it does not include assessment of skeletal muscle mass, which is increasingly seen as the primary indicator of cachexia in the current literature. More and more studies use CT images for the quantification and observation of muscle wasting in cancer cachexia and have shown a specific association between muscle loss and reduced survival [161, 171]. To conduct comparable clinical trials in the future, a clear definition of criteria for cachexia is indispensable.

### Translation of findings from animal models to human cancer cachexia therapy

Even though animal models are not sufficient to mimic all complex aspects of cachexia in cancer patients, results from these pre-clinical studies have already led to a substantial number of potential therapeutic targets and approaches.

#### Anti-cytokine treatments

In most animal studies, serum elevation of pro-inflammatory cytokines like TNF-α and IL-6 is associated with muscle wasting and anti-cytokine treatments showed great promise. Unfortunately, in humans the results from investigations of the role of cytokines are completely inconsistent. Most clinical trials of inhibitors of synthesis or activity of TNF-α have so far not proven to be effective in preserving lean body mass in cancer patients [9, 14, 20]. Thalidomide, an inhibitor of TNF-α and other pro-inflammatory cytokines, was shown to be effective in the treatment of cancer cachexia in patients with GIT cancers but has strong adverse side effects [161, 172–174]. Anti-TNF-α antibodies such as infliximab and etanercept did not show any significant improvements in cachectic patients and were not well tolerated either [175, 176].

Preclinical and clinical (phase I and II) studies performed on the IL-6 antibody ALD518 in patients with non-small cell lung cancer (NSCLC) showed that this treatment has the potential to improve anemia, reduce cancer-related cachexia and ameliorate fatigue, while having minimal adverse effects [173, 177]. Other anti-IL-6 antibodies or inhibitors (BMS 945429, selumetinib) have also been shown to be well tolerated and improve fatigue and loss of lean body mass [177, 178].

Currently, no anti-cytokine therapies are approved for the treatment of cancer cachexia and further clinical trials are needed to confirm their benefits. However, some clinical studies have shown that anti-cytokine treatments have the potential to ameliorate combination therapy protocols [9, 12].

The discrepancies between animal and human studies could be partly due to differences and difficulties in measuring serum cytokine levels or simply reflect the heterogeneity of the individual cytokine response in different types of cancers and patients [16]. Furthermore, pro-inflammatory mediators (especially IL-1) not only take effect in the inflammatory reaction but also have central effects leading to reduced food intake and anorexia in a complex and individual interaction with various hormones and neuropeptides. Another recently identified source of these discrepancies could be that different C26 tumor cell lines secret different amounts of IL-6 depending on sample storage conditions and the number of cell passages in vitro [179]. These results were reproduced in vivo and could implicate that measurements of IL-6 secretion in human cancer cachexia samples from different laboratories might largely vary depending on the treatment and storage conditions of samples.

#### Myostastin/ActRIIB targeting treatments

Blocking of myostatin and ActRIIB signaling showed very promising results in animal and in vitro studies. Several clinical approaches are currently being evaluated by pharmaceutical companies, but results are still lacking [180]. In cachexia due to heart failure, myostatin expression has been shown to be upregulated in animals [181] and patients [182]. In addition, increased myostatin expression has repeatedly been seen in cachectic patients secondary to HIV/AIDS or severe COPD [60]. Furthermore, mice with a heart-specific knockout of the myostatin gene appear to be resistant to skeletal muscle loss, which indicates that targeting this pathway could be of benefit for patients with muscle wasting in the context of chronic heart failure and maybe also in cancer cachexia [183, 184]. ActRIIB receptor and myostatin inhibitors are currently being evaluated in pre-clinical trials of muscle wasting and degenerative disorders. Among the first agents developed for clinical settings are the monoclonal anti-myostatin antibodies, which are currently undergoing phase II trials in patients with NSCLC and pancreatic cancer [161].

#### PIF/AngII targeting treatments

Proteolyis inducing factor (PIF) and AngII are important cachectic factors in experimental cancer wasting as well. In humans, however, the existence of a homologue to the murine PIF is still debated. Wieland et al. questioned the existence of human PIF after finding neither a correlation between the presence of this protein in urine and weight loss or survival of cancer patients, nor a specificity for malignant diseases, since they found the same protein in patients with cachexia due to congestive heart failure [89]. However, their study was later criticized for its methodology, since a cross-reactive antibody was used and western blot bands were not correctly confirmed [185]. In the end, results point towards a role of PIF, especially in GIT cancer, but its relevance still remains to be confirmed, and the source and mode of action of this protein need to be determined. Concerning the role of AngII in the pathophysiology of cancer cachexia, there are currently no published studies on human material. However, treatment with ACE inhibitors was found to ameliorate cardiac cachexia, and there are reports of unpublished results from clinical trials started by pharmaceutical companies which point to a possible benefit of this treatment in cancer cachexia [102]. This hypothesis should be confirmed in randomized controlled trials.

#### Treatments targeting increased proteolysis and decreased protein synthesis

Components of proteolytic systems were activated in most experimental models of cancer cachexia. However, in cancer patients these observations were not consistently confirmed, suggesting that different types of tumors and individual hosts may produce different reactions of the proteolytic systems. However, current attempts to pharmaceutically block the enhanced activity of the UPS by using antagonists of the inducers of proteasome expression, inhibitors of NF-κB signaling and inhibitors of ubiquitin ligases or proteasome subunits have not yet yielded any approved treatment options [99, 186]. Inhibitors of NF-κB, such as resveratrol, thalidomide, ibuprofen, eicosapentaenoic acid, and beta-hydroxy-beta-methylbutyrate, have been shown to improve skeletal muscle mass in cachexia. In addition, proteasome inhibitors have been shown to have positive effects in Duchenne and Becker muscular dystrophy. However, in a study on patients with pancreatic cancer, treatment with bortezomib showed no beneficial effects on cachexia [187].

There is still much debate over whether the predominant component of muscle loss during cachexia is increased proteolysis or decreased protein synthesis. However, the anabolic Akt/mTOR pathway was identified as the most important anabolic cascade, and cross-talk with proteolytic pathways was demonstrated in animal models. However, some studies found protein synthesis to be activated rather than suppressed. This might reflect the fact that cachexia is a dynamic process, passing through different stages that might be dominated either by protein breakdown or protein synthesis. For example, it is possible that during certain stages of cachexia, protein synthesis is actually increased in skeletal muscle due to the local production of cytokines or as a counter-regulatory phenomenon. In this context, it becomes clear why it is hard to interpret human studies on cancer cachexia, which usually include heterogeneous subjects in terms of the stage of cachexia. To overcome this problem it is essential that future studies accurately define different stages of cachexia so their results can be stratified accordingly.

The impairment of muscle regeneration is a relatively new aspect of muscle loss in cancer cachexia and, as presented above, data on its role in human cancer cachexia remains very limited. To our knowledge no drug targets have been preselected in this field and further research is needed to identify and test those.

#### Current cancer cachexia therapy options

Considering the multidimensional background of cancer cachexia, it is more and more the accepted view that multimodal therapeutic approaches, including exercise, nutrient supplementation, appetite stimulation and pharmacological intervention, have to be implemented and individually tailored for patients at different stages of cachexia [5, 161]. A large-scale meta-analysis showed that nutritional interventions were successful in increasing energy intake, body weight and some aspects of QoL [188]. The evidence for interventions with resistance exercise training is not as extensive yet, but first results are promising [189]. Nutrient supplementation with N3-fatty acids, e.g. eicosapentaenoic acid or fish oil, also have shown positive effects on muscle loss and survival; however, the evidence is not yet sufficient for recommendation [161]. In addition, improving patients’ metabolism by insulin or metformin treatment was shown to increase whole body fat (without counteracting muscle loss) and survival in initial study results [161, 190]. Moreover, secondary symptoms like pain, diarrhea or stomatitis have to be managed correctly to evaluate the efficacy of new treatments of cancer cachexia [161, 164]. Additional multidimensional pharmacological therapy should ideally include drugs that target the inflammatory status, oxidative stress, nutritional disorders, muscle catabolism, anemia, immunosuppression, and fatigue [173]. Anti-inflammatory drugs like COX inhibitors (indomethacin, ibuprofen) not only reduce the inflammatory response but also have a positive effect on REE and were shown to prolong survival in malnourished patients with advanced cancer [191]. Finally, careful psychosocial counseling and access to self-help groups should be provided [173]. Moreover, successful surgical removal of the tumor and/or oncological treatments should be the starting point for rehabilitation of patients with cancer-associated muscle wasting [160].

## Conclusion

In conclusion, given the heterogeneous and multi-factorial etiology of cachexia, it is likely that this syndrome is a result of deregulation of multiple signaling pathways. It is possible that certain pathways are involved only in a subset of patients and to an individual extent, which would determine if the patient responds to therapeutic interventions on the level of intracellular signaling pathways. However, there is also a belief that treatments that preserve muscle mass *per se* can improve survival and QoL of cancer patients, regardless of the underlying molecular mechanisms. This review shows that, even though animal models cannot imitate all of the complex aspects of human cancer cachexia, they provide a robust setting to develop and test new, targeted therapies. Ultimately, further studies on the signaling pathways that lead to skeletal muscle loss in larger and more homogeneous cohorts of human patients are warranted to confirm potential drug targets identified in experimental animal models.

## Abbreviations

GIT: gastrointestinal tract
COPD: chronic obstructive pulmonary disease
QoL: quality of life
BMI: body mass index
CRP: C-reactive protein
REE: resting energy expenditure
ATP: adenosin-triphosphate
UCP: uncoupling protein
TNF-α: tumor necrosis factor-alpha
LLC: Lewis lung cell carcinom
TRAF-6: TNF-receptor adaptor protein
TNFR: TNF-receptor
mRNA: messenger ribonucleic acid
NF-κB: nuclear factor κB
IL: interleukin
NSCLC: non-small cell lung cancer
INF-γ: interferon gamma
VEGF: vascular endothelial growth factor
TGF: transforming growth factor
GDF: growth differentiation factor
ActRIIB: activin type-2 receptor
IGF: insulin-like growth factor
MIC: macrophage inhibitory cytokine
PIF: proteolysis-inducing factor
UPS: ubiquitin proteasome system
PKR: RNA-dependent protein kinase
HCAP: human cachexia-associated protein
Ang II: angiotensin-II
REE: resting energy expenditure
ROS: reactive oxygen species
ACE: angiotensin-converting enzyme
MuRF-1: muscle ring finger-1
MAfxb: muscle atrophy F box
Atgs: autophagy related genes
ALS: authophagy-lysosomal system
GSK: glycogen synthase kinase
mTOR: mammalian target of rapamycin
MRF: muscle growth and regeneration factor
CT: computed tomography
HIV/AIDS: human immunodeficiency virus/ acquired immune deficiency syndrome
COX: cyclooxygenase
